# Effects of Masks Worn to Protect Against COVID-19 on the Perception of Facial Attractiveness

**DOI:** 10.1177/20416695211027920

**Published:** 2021-06-27

**Authors:** Miki Kamatani, Motohiro Ito, Yuki Miyazaki, Jun I. Kawahara

**Affiliations:** Hokkaido University, Sapporo, Japan; Japan Society for the Promotion of Science, Tokyo, Japan; The University of Tokyo, Japan; Fukuyama University, Japan; Hokkaido University, Sapporo, Japan

**Keywords:** sanitary mask, COVID-19, facial attractiveness, healthiness

## Abstract

Wearing a sanitary mask tended, in the main, to reduce the wearer’s sense of perceived facial attractiveness before the COVID-19 epidemic. This phenomenon, termed *the sanitary-mask effect*, was explained using a two-factor model involving the occlusion of cues used for the judgment of attractiveness and unhealthiness priming (e.g., presumed illness). However, these data were collected during the pre-COVID-19 period. Thus, in this study, we examined whether the COVID-19 epidemic changed the perceived attractiveness and healthiness when viewing faces with and without sanitary masks. We also used questionnaires to evaluate beliefs regarding mask wearers. We found that the perception of mask-worn faces differed before versus after the onset of the COVID-19 epidemic. Specifically, mask-wearing improved wearers’ sense of the attractiveness of faces, which were rated as less attractive when a mask was not worn after the onset of the COVID-19 epidemic. Furthermore, mask-worn faces were rated as healthier after the onset of the COVID-19. The proportion of respondents with negative associations regarding mask-wearing (e.g., unhealthiness) decreased relative to before the epidemic. We suggest that the weakening of this association altered the sanitary-mask effect with a relative emphasis on the occlusion component, reflecting the temporal impact of a global social incident (the COVID-19 epidemic) on the perception of facial attractiveness.

The COVID-19 epidemic has altered daily life worldwide. In affected areas, individuals attempt to maintain a physical distance between themselves and others, wash their hands frequently, and wear sanitary masks in workplaces and public spaces. Authorities have encouraged citizens to wear sanitary masks in public environments (e.g., World Health Organization, 2020), leading to a large increase in the number of people who routinely wear sanitary masks compared to before the COVID-19 epidemic (Chen et al., 2020). The necessity to avoid COVID-19 infection has motivated people to wear masks despite certain perceptual side effects. These include increased difficulty in the identification of individuals (Carragher & Hancock, 2020; Noyes et al., 2021) and recognition of facial expressions due to partial occlusion of the face by a mask (Grundmann et al., 2021; Noyes et al., 2021; Zhang et al., 2018).

Wearing a sanitary mask can affect the perception of facial attractiveness. A study conducted before the COVID-19 epidemic demonstrated that wearing a sanitary mask can decrease the externally rated facial attractiveness of the wearer (the sanitary-mask effect; Miyazaki & Kawahara, 2016). The researchers proposed a two-factor model explaining the sanitary-mask effect and argued that both the effect of occlusion and priming regarding unhealthiness are implicated in the phenomenon (Figure 1). The first factor in the model was the effect of occlusion, specifically, that of the lower part of the face by a sanitary mask. Such occlusion can reduce informative features, such as the symmetry and contours of facial structures, the smooth/roughness of the skin, and skin color. These features contribute to the perception of facial attractiveness. For example, asymmetric facial contours, as well as misaligned or distorted facial features, generally reduce the perceived attractiveness of a face (e.g., Little & Jones, 2003; Rhodes et al., 1998, 1999; Scheib et al., 1999). Similarly, pimples and scars can reduce perceived attractiveness (e.g., Jaeger et al., 2018). The visual occlusion of such unfavorable features by a mask could increase the ratings of attractiveness for faces that would otherwise receive low attractiveness ratings. By contrast, faces rated as highly attractive often have symmetric contours with no distortions, as well as smooth skin. Occlusion of these favorable features by a mask could decrease perceived attractiveness. The second factor in the two-factor model was priming regarding unhealthiness. Specifically, during the pre-COVID-19 period in Japan, a mask could indicate that the wearer had a medical condition (e.g., illness or respiratory allergy). Thus, a sanitary mask could invoke the impression that the mask wearer was unhealthy and/or vulnerable. Perceptions of ill health can negatively impact perceived facial attractiveness (Jones et al.,2004). Therefore, regarding priming for unhealthiness, the perceived attractiveness of a mask-worn face would be expected to decrease regardless of the level of facial attractiveness when masks were not worn.

**Figure 1.**
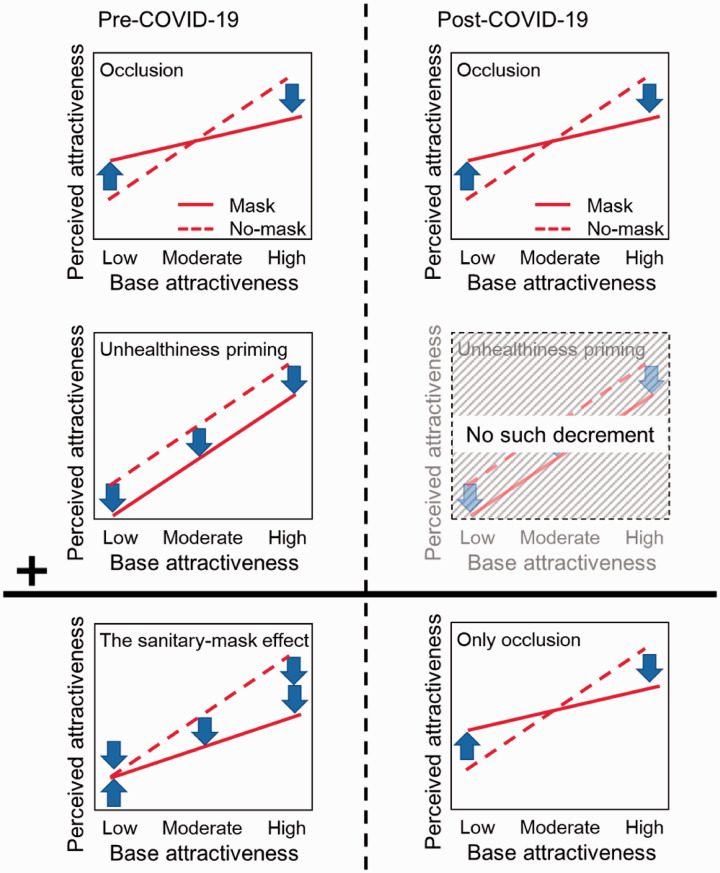
The left column represents the sanitary-mask effect. The right column represents the main hypothesis of this study, that is, that the association between sanitary mask wearing and perceived unhealthiness would disappear during the COVID-19 epidemic.

Overall, the model predicts that the perceived attractiveness of mask-worn faces would be greatly reduced for faces with high levels of baseline attractiveness because of the combined effects of occlusion and unhealthiness priming. By contrast, the model predicts that perceived attractiveness would remain unchanged for faces with low baseline attractiveness ratings because of an interaction between the effects of occlusion (i.e., concealment of disadvantageous features) and unhealthiness. This model was supported by the finding that the interaction between the two factors disappeared when the lower area of the face was covered by an object unrelated to health, such as a notebook or a piece of paper, such that only the effect of occlusion remained (Miyazaki & Kawahara, 2016). In other words, unhealthiness priming only occurred when part of the face was occluded by a sanitary mask.

Because the abovementioned sanitary-mask effect was described prior to the COVID-19 epidemic, it is reasonable to assume that the effect might have changed as people’s attitudes change in response to recent events. For example, Rudman et al. (2013) found that people’s attitudes toward politicians who were concerned about climate change improved following hurricanes in 2011 and 2012 that caused destructive damage in the United States. Similarly, Siegrist and Visschers (2013) found that the accident at the Fukushima Daiichi Nuclear Power Plant adversely affected the acceptance of nuclear power in the German-speaking region of Switzerland, based on surveys conducted before, immediately after, and at 6 months after the accident. Therefore, the COVID-19 epidemic, which is one of the largest global health emergencies in recent history, might have also impacted people’s attitudes toward sanitary mask wearers and mask-worn faces. The purpose of wearing a sanitary mask has changed from personal medical conditions (e.g., illness or respiratory allergies) to the mutual protection of community members from COVID-19 viral spread and infection, as well as compliance with social norms. Indeed, seeing other people wear masks during the COVID-19 epidemic is likely to encourage individuals to wear masks (Nakayachi et al., 2020). Thus, we expected that attitudes regarding sanitary mask wearing would differ before versus after the onset of the COVID-19 epidemic.

We conducted three studies to examine perception regarding sanitary mask wearing. In Study 1, we investigated whether the COVID-19 epidemic positively altered belief regarding sanitary mask wearing by comparing data obtained in the pre-COVID-19 period with current data. We included mask color as a factor (white vs. black) because black tends to be more strongly associated with negative concepts compared to white (Lakens et al., 2013; Meier et al.,2004; Sherman & Clore, 2009; Specker et al., 2018). Moreover, perceptions of wearers of black sanitary masks were more negative compared with those of wearers of white sanitary masks during both the pre- and post-COVID-19-onset periods in Japan (Ito & Kawahara, 2019; Kamatani et al., in press). However, the association between black sanitary masks and unhealthiness would be weakened after the onset of the COVID-19 epidemic.

Because the purpose of mask-wearing in Japan has changed before versus after the onset of the COVID-19 epidemic, we predicted that beliefs regarding sanitary mask wearers would have shifted in a positive direction compared with during the pre-COVID-19 period, regardless of the mask color. In Study 2, we measured the perceived attractiveness of mask-worn faces. Specifically, we readministered the survey and rating task from Miyazaki and Kawahara (2016). We predicted that the sanitary-mask effect on the perceived attractiveness of mask-worn faces would have changed because of the COVID-19 epidemic. Specifically, we predicted that the epidemic would have reduced unhealthiness priming for sanitary masks, such that the effect of mask-wearing would be caused solely by occlusion of the face. In Study 3, we measured the perceived healthiness of wearers of white and black masks, using the procedures from Miyazaki and Kawahara (2016). We predicted that the mask-worn faces would be perceived as healthier after versus before the onset of the COVID-19 epidemic, because the epidemic would have altered the purpose of sanitary mask wearing, thus weakening the association between unhealthiness and sanitary masks.

## Study 1: Beliefs Regarding Wearers of Sanitary Masks

We investigated whether beliefs regarding the attractiveness and healthiness of wearers of white sanitary masks became more positive after versus before the onset of the COVID-19 epidemic. We also examined beliefs regarding wearers of black masks, which were strongly negative before the epidemic (Ito & Kawahara, 2019). We predicted that these negative beliefs would be weakened after the onset of the epidemic. Because the COVID-19 epidemic increased the prevalence of mask-wearing behavior, we also expected the association between sanitary mask wearers and unhealthiness to have weakened.

### Methods

#### Participants

We recruited 286 adults (153 males and 133 females; *M* age = 20.05 years, *SD* = 2.27) to participate in a survey regarding the facial attractiveness of women who were wearing sanitary masks. The number of participants was identical to that in Miyazaki and Kawahara (2016). In this and the following studies in this article, all participants provided informed consent and participated in exchange for course credits or a monetary reward. The experimental protocol was approved by the ethical review board of Hokkaido University.

#### Procedure

The survey was administered using Google Forms. The participants were asked to use a seven-point Likert-type scale to report their beliefs regarding women wearing white or black sanitary masks in terms of attractiveness and healthiness. They were not shown any images. The survey consisted of the following four items: (a) “Is the facial attractiveness of a woman increased (or decreased) when she wears a white sanitary mask?”; (b) “Is the facial attractiveness of a woman increased (or decreased) when she wears a black sanitary mask?”; (c) “What do you think about a person who is wearing a white sanitary mask?”; and (d) “What do you think about a person who is wearing a black sanitary mask?.” These items were consistently presented in this order on the same page on a computer screen. Data were collected from June 26, 2020 to December 4, 2020, that is, during the COVID-19 epidemic.

#### Statistical Analysis

The responses regarding attractiveness were arranged into three categories: negative responses, neutral responses, and positive responses. Responses ranging from 1 = *greatly decreased* to 3 = *slightly decreased* were pooled as negative responses. Similarly, responses ranging from 5 = *slightly increased* to 7 = *greatly increased* were pooled as positive responses. The response 4 = *no change* was treated as a neutral response. The same pooling scheme was applied to the responses on healthiness. A chi-square test and residual analyses (Haberman, 1973) were conducted to compare the pre-COVID-19 data (i.e., Miyazaki & Kawahara, 2016; *N* = 286) with those collected after the onset of the COVID-19 epidemic (i.e., the present study) using R software (version 3.6.1).

### Results

**Figure 2.**
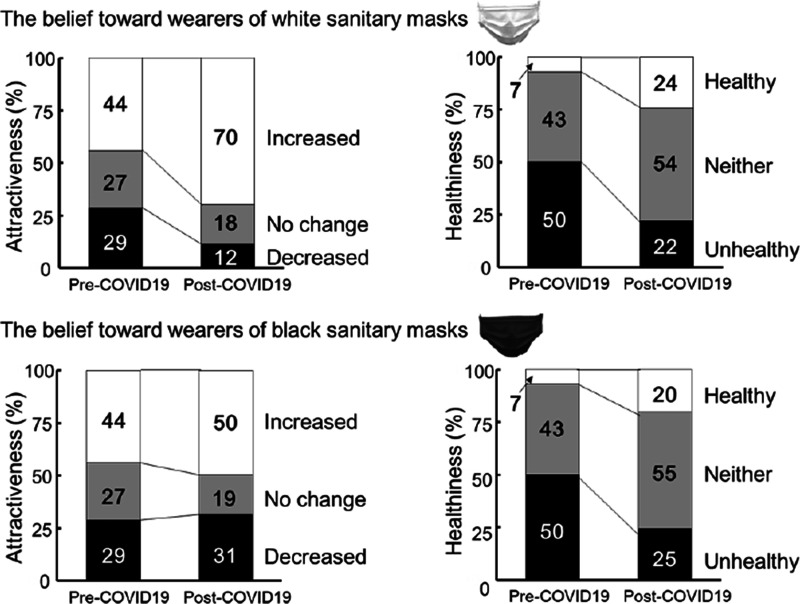
Stacked column charts showing the percentages of perceived attractiveness and healthiness scores during the pre- and post-COVID-19-onset periods (upper row: ratings for faces wearing white sanitary masks; bottom row: pre-COVID-19 ratings were for white masks only and post-COVID-19-onset ratings for black masks only). The pre-COVID-19 data were taken from Miyazaki and Kawahara (2016).

Figure 2 shows the percentages of responses regarding attractiveness and healthiness for white (top row) and black (bottom row) masks for each item, presented as stacked column charts. The left column in each panel represents pre-COVID-19-onset data and the right shows post-COVID-19-onset data. Regarding the scores for white sanitary masks, we identified significant effects of period (pre- vs. post-COVID-19 onset) on healthiness and attractiveness ratings—healthiness: χ^2^ (2, *N* = 572) = 61.515, *p* < .001, *V* = .328; attractiveness: χ^2^ (2, *N* = 572) = 42.447, *p* < .001, *V* = .272. Residual analyses of the healthiness data revealed that the number of people who answered *healthy* or *neither* increased (*p*s < .001), whereas the number of people who answered *unhealthy* decreased (*p* < .001) compared to the pre-COVID-19 period. In terms of attractiveness, more people answered that mask-wearing *increased the level of attractiveness* (*p* < .001), whereas fewer people answered that mask-wearing led to *no change* or *decreased the level attractiveness* (*p*s < .012) compared to the pre-COVID-19 period.

Regarding the scores for black sanitary masks, we identified a significant effect of period (pre- vs. post-COVID-19 onset) on healthiness and a trend for the effect of period on attractiveness—healthiness: χ^2^ (2, *N* = 572) = 47.891, *p* < .001, *V* = .289; attractiveness: χ^2^ (2, *N* = 572) = 5.691, *p* = .058, *V* = .100. Residual analyses were conducted for the healthiness ratings. More people answered *healthy* or *neither* post-COVID-19-onset (*p*s < .003), whereas fewer people answered *unhealthy*(*p* < .001) compared to the pre-COVID-19 period.

### Discussion

The results indicate that the beliefs toward sanitary mask wearers during the post-COVID-19-onset period differed from those during the pre-COVID-19 period. The number of respondents who reported that they thought mask wearers were unhealthy decreased, regardless of the color of the mask. Instead, the number of respondents who reported that they felt neutral or that they thought mask wearers were healthy increased. Thus, beliefs regarding mask wearers in terms of healthiness shifted toward a positive direction. Regarding attractiveness, more respondents reported that wearing a white sanitary mask increased the user’s attractiveness, whereas fewer respondents reported that wearing a white mask led to no change or a decrease in attractiveness. The ratings for black sanitary mask wearers were comparable both before and after the onset of the COVID-19 epidemic.

## Study 2: Perceived Attractiveness of Mask-Worn Faces

Here, we examined the impact of the COVID-19 epidemic on the perceived attractiveness of mask-worn faces. Specifically, we measured perceived attractiveness using the same stimuli and procedure employed by Miyazaki and Kawahara (2016, Experiment 1). We predicted that the sanitary-mask effect on perceived attractiveness of mask-worn faces would be altered by COVID-19 because the epidemic reduced the degree to which sanitary masks might prime for unhealthiness, leaving just the effect of occlusion. Accordingly, we expected the perceived attractiveness of faces with low attractiveness ratings to increase when wearing a mask and anticipated that the opposite would be true for faces with high attractiveness ratings (Figure 1, bottom right). In other words, we expected the function of perceived attractiveness for no-mask and mask-worn faces to intersect at the moderate level of baseline attractiveness.

### Methods

#### Participants

Fifty-nine undergraduate and graduate students (29 males and 30 females; *M* age = 19.98 years, *SD* = 1.61) participated in Study 2. All participants reported normal or corrected-to-normal eyesight and color vision and were recruited from a participant pool at Hokkaido University.

#### Apparatus and Stimuli

Study 2 was conducted from May 19, 2020 to July 30, 2020. The stimuli were presented on a web browser on each participant’s personal computer, controlled by lab.js software (Henninger et al., 2020). No participants completed the experiment using their smartphone.

The stimuli were the same as those used in Miyazaki and Kawahara (2016). They consisted of 66 images chosen from a homemade database of young Japanese female faces (Kawahara & Kitazaki, 2013). The facial images (354 × 472 pixels each, with a height of 6.85 cm and width of 9.10 cm on a 14-in. laptop screen) had been prerated in terms of facial attractiveness (0: *less attractive*, 100: *highly attractive*). Of the 66 facial images, 22 were categorized as having low levels of attractiveness (*M* = 20.29, *SD* = 2.66), 22 as having moderate levels of attractiveness (*M* = 38.01, *SD* = 0.70), and 22 as having high levels of attractiveness (*M* = 58.72, *SD* = 2.86). The faces expressed a slight smile or had a neutral expression. None of the facial images showed a person wearing glasses.

To create an image of a face with a white sanitary mask, an image of a white mask was superimposed onto the chosen facial image using a graphic editor. Unnatural edges between the face and the mask caused by the superimposing procedure were removed using a Gaussian blur effect in the graphic editor (Adobe Photoshop CS6). We also created images of faces with a black sanitary mask using the same procedure and graphic editor. The image generation procedure was similar to that used in Miyazaki and Kawahara (2016). Of the 22 images in each attractiveness category, half consisted of faces wearing sanitary masks (white or black) and the other half consisted of faces without masks.

#### Procedure

At the beginning of each trial, a single facial image was presented at the center of the screen accompanied by a horizontal rating scale located below the image. The participants were asked to rate the attractiveness of the face from 1 (非常に魅力的でない: *very unattractive*) to 100 (非常に魅力的である: *very attractive*) by moving the slider on the rating scale. They clicked on the *submit* button to report their scores. As mentioned, the participants were shown 66 facial images, half with and half without masks, from three sets of images (high, moderate, and low levels of attractiveness, 22 images from each set). Half of the 22 images showed a mask-worn face, and the other half showed a no-mask face. Participants never saw the same face. The presence or absence of masks was counterbalanced by identity across participants. After a trial was complete, a blank screen was shown for 500 ms before the next trial began. The presentation order of the images was randomized across participants. The mask color was consistent for each participant and was blocked as a between-subjects factor.

### Results

Before directly comparing the pre-COVID-19 data (i.e., Miyazaki & Kawahara, 2016; *N* = 29) with those collected after the onset of the COVID-19 epidemic (i.e., the present study), we examined whether the baselines (i.e., attractiveness ratings of no-mask faces) of the two studies were similar. We concluded that a direct comparison was inappropriate because the baselines of the two studies differed due to an interaction between period and baseline attractiveness. Specifically, a two-way (Period × Baseline attractiveness) analysis of variance of rating scores for no-mask faces indicated significant main effects for both period—*F* (1, 86) = 5.036, *p* =.027, ${\text{η}}_{\text{p}}^{\text{2}}$ = .055) and baseline attractiveness—*F* (2, 172) = 636.749, *p* < .001, ${\text{η}}_{\text{p}}^{\text{2}}$ = .881. The interaction between period and baseline attractiveness—*F* (2, 172) = 586.657, *p* < .001, ${\text{η}}_{\text{p}}^{\text{2}}$ = .096—was significant. Therefore, we analyzed the post-epidemic results separately from the pre-epidemic results.

**Figure 3.**
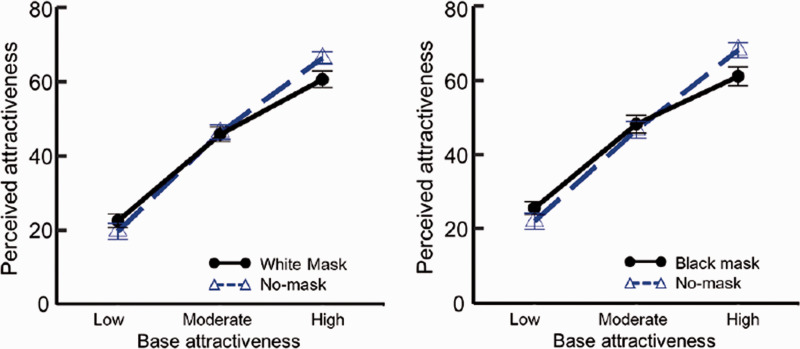
Results of Study 2. Mean levels of perceived attractiveness as a function of mask-wearing and baseline attractiveness (left: perceived attractiveness of faces with white masks or no masks; right: perceived attractiveness of faces with black masks or no masks). Error bars represent standard errors.

The mean rating scores were plotted as a function of baseline attractiveness for both mask-worn (solid line) and no-mask (dashed line) faces, as shown in Figure 3. We conducted a three-way (Baseline attractiveness × Mask presence × Color) mixed design analysis of variance with color as a between-subjects factor and mask-wearing and baseline attractiveness as within-subject factors. We identified a significant main effect of baseline attractiveness—*F* (2, 114) = 455.147, *p* < .001, ${\text{η}}_{\text{p}}^{\text{2}}$ = .888—indicating that the attractiveness increased linearly with baseline attractiveness (Holm’s test: *t*s (57) > 15.465 *p*s <.001, *r*s > .89). No significant main effects from mask-wearing or mask color were found—mask-wearing: *F* (1, 57) = 0.455, *p* = .502, ${\text{η}}_{\text{p}}^{\text{2}}$ = .007; color: *F* (1, 57) = 0.734, *p* = .395, ${\text{η}}_{\text{p}}^{\text{2}}$ = .012. Importantly, the interaction between baseline attractiveness and mask condition was significant—*F* (2, 114) = 25.221, *p* < .001, ${\text{η}}_{\text{p}}^{\text{2}}$ = .306. Multiple comparisons of this interaction revealed that faces wearing masks were perceived as less attractive than those without masks for faces with high attractiveness scores—*F* (1, 57) = 15.420, *p* < .001, ${\text{η}}_{\text{p}}^{\text{2}}$ = .212. By contrast, faces wearing masks were perceived as more attractive than those without masks for faces with low attractiveness scores—*F* (1, 57) = 7.360, *p* = .008, ${\text{η}}_{\text{p}}^{\text{2}}$ = .114. For faces with moderate attractiveness scores, the difference in perceived attractiveness between the mask-worn and no-mask conditions was not significant—*F* (1, 57) = 0.061, *p* = .804, ${\text{η}}_{\text{p}}^{\text{2}}$ = .001.

Although an analysis including the Period × Baseline attractiveness × Mask presence interaction would be optimal for this study due to the difference in baseline, we report this for fairness. A three-way (Period × Baseline attractiveness × Mask presence) analysis of variance of rating scores revealed a significant main effect of baseline attractiveness—*F* (1, 172) = 632.291, *p* < .001, ${\text{η}}_{\text{p}}^{\text{2}}$ = .880. No main effects of period or mask presence were found—Period: *F* (1, 86) = 2.502, *p* = 0.117, ${\text{η}}_{\text{p}}^{\text{2}}$ = .028; Mask presence: *F* (1, 86) = 3.870, *p* = .052, ${\text{η}}_{\text{p}}^{\text{2}}$ = .043. The interactions were not significant—Period × Mask presence: *F* (1, 86) = 2.379, *p* = .126, ${\text{η}}_{\text{p}}^{\text{2}}$ = .026; Period × Baseline attractiveness × Mask presence: *F* (1, 172) = 0.364, *p* = .695, ${\text{η}}_{\text{p}}^{\text{2}}$ = .004.

### Discussion

In this study, we identified a critical interaction that supports the two-factor model of the sanitary-mask effect, although the colors of the masks did not affect the attractiveness ratings. Mask-worn faces were perceived as more attractive than no-mask faces when the baseline attractiveness was low. The opposite was also true, such that mask-worn faces were perceived as less attractive than no-mask faces when the baseline attractiveness was high. This is consistent with the finding by Miyazaki and Kawahara (2016) regarding faces occluded by objects unrelated to health status. These results are also consistent with the prediction of the model (Figure 1, right column).

The model indicates that the sanitary-mask effect occurs in conjunction with the loss of critical cues due to occlusion of the lower face and mask-related unhealthiness priming. Because the data in Study 2 were obtained after the onset of the COVID-19 epidemic, the association between mask-wearing and unhealthiness had weakened. Therefore, as unhealthiness priming no longer reduced the perceived attractiveness of mask-worn faces, attractiveness was modulated solely by visual occlusion. Thus, mask-worn faces in the low-attractiveness group were perceived as more attractive and mask-worn faces in the high-attractiveness group were perceived as less attractive compared with the baseline attractiveness ratings.

The effect of mask color was minimal. A previous study demonstrated that wearers of black masks were perceived as less attractive compared to wearers of white masks when the baseline attractiveness was high (Ito & Kawahara, 2019; Kamatani et al., in press). We found no such effect in this study.

## Study 3: Perceived Healthiness of Mask-Worn Faces

We examined the impact of the COVID-19 epidemic on the perceived healthiness of mask-worn faces using the experimental methods from Miyazaki and Kawahara (2016, Experiment 5). We predicted that mask-worn faces would be perceived as less unhealthy during the COVID-19 period compared with before the epidemic. Because the epidemic changed the purpose of sanitary mask wearing—from personal medical conditions (e.g., coughing) to engagement in society-wide prevention of COVID-19 infection—we expected the association between perception of unhealthiness and sanitary masks to be weakened, resulting in improved ratings of healthiness for mask-worn faces during the COVID-19 period.

### Methods

#### Participants

Forty-four people (23 males and 21 females; *M* age = 41.18 years, *SD* = 9.98) participated in Study 3. The participants were recruited using CrowdWorks, a crowdsourcing service. Study 3 was conducted on January 15, 2021. The instructions and stimuli were presented on a web browser on the participant’s personal computer, controlled by lab.js software (Henninger et al., 2020). Responses were recorded by clicking a mouse or touchpad. No participants completed the experiment using their smartphone. The apparatus and stimuli were same as in Study 2.

#### Procedure

The stimuli and procedures were identical to those of Study 2 except that participants rated the healthiness of facial images from 1 (非常に健康的でない: *very unhealthy*) to 100 (非常に健康的である: *very healthy*) by moving the slider on a rating scale presented below the face image. They clicked the *submit* button to report their scores. Because we observed no effect of mask color in Study 2, we collapsed the factor of mask color (white and black) in this study, although the factor was included as a hidden variable in the between-subjects analysis.

### Results

**Figure 4.**
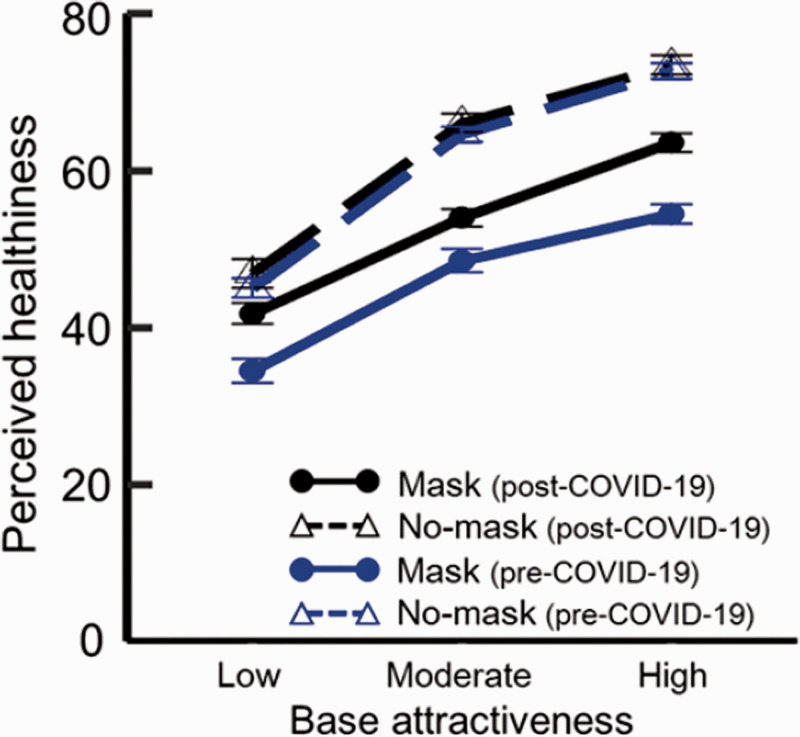
Results of Study 3. Mean levels of perceived healthiness as a function of baseline attractiveness under with mask and no-mask conditions. Error bars represent standard errors.

The mean rating scores were plotted as a function of baseline attractiveness for mask-worn (solid line) and no-mask (dashed line) faces under the pre- (blue line) and post-COVID-19-onset (black line) conditions, as shown in Figure 4. To examine the effect of period (pre- vs. post-COVID-19 onset), we compared the present data with the healthiness rating data of Miyazaki and Kawahara (2016; *N* = 26) and conducted a three-way (Period × Baseline attractiveness × Mask presence) mixed design analysis of variance of rating scores and their counterparts with period as the between-subjects factor and with mask presence and baseline attractiveness as within-subject factors. We also conducted simple effect tests and Holm’s test. We identified significant main effects of period, mask-wearing, and baseline attractiveness—Period: *F* (1, 68) = 6.097, *p* = .016, ${\text{η}}_{\text{p}}^{\text{2}}$ = .082; Baseline attractiveness: *F* (2, 136) = 273.266, *p* < .001, ${\text{η}}_{\text{p}}^{\text{2}}$ = .800; Mask-wearing: *F* (1, 68) = 71.164, *p* < .001, ${\text{η}}_{\text{p}}^{\text{2}}$ = .511. The significant period effect indicated that the perceived healthiness was greater during versus before the COVID-19 epidemic. The significant mask effect indicated that mask-worn faces were perceived as less healthy than faces without masks. The significant baseline-attractiveness effect indicated that the baseline attractiveness increased linearly (*t*s (68) > 8.769, *p* < .001, *r*s >.72). Importantly, the interaction between period and mask presence was significant—*F* (1, 68) = 4.669, *p* = .034, ${\text{η}}_{\text{p}}^{\text{2}}$ = .064. Multiple comparisons for this interaction revealed that mask-worn faces were perceived as healthier during the COVID-19 epidemic relative to the pre-COVID-19 period—*F* (1, 68) = 10.313, *p* = .002, ${\text{η}}_{\text{p}}^{\text{2}}$ = .131. However, this was not the case for no-mask faces—*F* (1, 68) = 0.376, *p* = .541, ${\text{η}}_{\text{p}}^{\text{2}}$ = .005. The interaction between baseline attractiveness and mask presence was also significant—*F* (2, 136) = 11.407, *p* < .001, ${\text{η}}_{\text{p}}^{\text{2}}$ . = 143. Multiple comparisons showed that mask-worn faces were perceived as less healthy than no-mask faces, regardless of baseline attractiveness—*F* (1, 68) > 17.870, *p* < .001, ${\text{η}}_{\text{p}}^{\text{2}}$ > .208. No other interactions were significant.

### Discussion

The results of Study 3 demonstrated that the perceived unhealthiness of mask-worn faces was lower after the onset of the COVID-19 epidemic compared with before the epidemic, consistent with our prediction. This reduction was likely due to changes in the perceived purposes of mask use. Specifically, whereas participants might have associated sanitary masks with personal medical conditions (e.g., coughing or respiratory allergies) before the epidemic, mask-wearing after the onset of the epidemic was more likely to be associated with the prevention of COVID-19 infection and compliance with social norms (Nakayachi et al., 2020).

These results were consistent with the findings from Study 1 in two aspects. First, the association between unhealthiness and mask-wearing weakened after the onset of the COVID-19 epidemic. Second, the reduction in the perception of unhealthiness occurred regardless of whether the respondents saw (Study 3) or merely imagined (Study 1) mask-worn faces. Given that the sanitary-mask effect on perceived attractiveness is determined by the interaction between occlusion and unhealthiness priming according to the two-factor model, a reduction in the negative impact of perceived unhealthiness (Studies 1 and 3) would leave occlusion to act as the primary or solo factor contributing to the perception of facial attractiveness. Therefore, the two-factor model can consistently explain the results of Study 2. Specifically, occlusion can hide favorable facial features on attractive faces and unfavorable features on less attractive faces, resulting in under- and overestimations of attractiveness for baseline attractive and unattractive faces, respectively.

## General Discussion

In this study, we investigated the impact of the COVID-19 epidemic on beliefs regarding sanitary mask wearers as well as the perceived attractiveness of mask-worn faces by comparing data collected pre- and post-COVID-19-onset in Japan. Study 1 revealed that beliefs regarding sanitary mask wearers during the COVID-19 period differed from those in the pre-COVID-19 period. Specifically, the number of respondents who reported that they felt mask wearers were unhealthy decreased regardless of the mask color. Instead, the number of respondents who rated mask wearers as neutral or healthy increased. This change in belief was strengthened by the disappearance of the sanitary-mask effect after the onset of the epidemic, as shown in Study 2. During the pre-COVID-19 period, mask wearers were perceived as less attractive in general. This indicates that the discount in perception of attractiveness caused by mask-wearing was larger for baseline attractive faces and smaller or negligible for baseline unattractive faces (Miyazaki & Kawahara, 2016). This discounted perception did not occur for baseline unattractive faces in this study. Instead, for mask-worn faces, the perceived attractiveness ratings for baseline unattractive faces were higher.

This change in the perceived attractiveness of mask-worn faces can be explained by the reduced association between unhealthiness and sanitary masks. This reduction was supported by the results of Study 3, that is, that mask-worn faces were perceived as healthier after the onset of the COVID-19 epidemic compared with before the epidemic. Mask-worn faces were perceived as less healthy than no-mask faces regardless of the measurement period (before or after the onset of the epidemic). However, our data indicate that the association between mask-wearing and unhealthiness had weakened. We suggest that this reduction in the strength of the association was caused by the change of the purpose of mask use. Specifically, before the COVID-19 epidemic, masks were associated with personal medical problems experienced by the wearer, such as symptoms of illness (e.g., coughing or rhinorrhea) or allergies to pollen. After the onset of the epidemic, masks became associated with society-wide attempts to prevent the spread of COVID-19 infection and have become a social norm such that seeing mask-worn people may encourage an individual to wear a mask (Nakayachi et al., 2020).

Our results were consistent with the two-factor model of the sanitary-mask effect. Miyazaki and Kawahara (2016) provided converging evidence to support the model, which was proposed before the COVID-19 epidemic. Furthermore, prior to the onset of the epidemic, they predicted that removing the perception of unhealthiness associated with mask-wearing would reduce the negative impact on attractiveness ratings. To examine this possibility, they replaced a mask with a notebook and found that the results supported their prediction. They replicated this finding by replacing a mask with a card that occluded the same lower area of the face. The pattern they observed was similar to our finding in Study 2, which was consistent with the two-factor model.

Our data, along with those of previous studies, indicate that the mechanism underlying the modulation of attractiveness by mask-wearing is related to the occlusion of critical features. Occluding less attractive faces can hide negative features, such as asymmetric contours, imbalanced arrangements of facial features, and pimples. This could shift attractiveness ratings toward the average, and thus improve ratings for baseline unattractive faces. The opposite is true for attractive faces. Occluding attractive faces can hide positive features, such as symmetric contours, balanced arrangements of features, and smooth skin. This could shift attractiveness ratings toward the average, thus reducing ratings for baseline attractive faces. These ideas were supported by the findings of Study 2. Because the association between unhealthiness and mask-wearing had weakened after the onset of the COVID-19 epidemic, the effects of masks on ratings of perceived unhealthiness were similar to those of the notebooks and cards used to occlude faces in Miyazaki and Kawahara’s (2016; Experiments 3a, 3b, and 4) occlusion experiments. This mechanism may be related to recent findings that perceived facial attractiveness ratings improved when faces were partially occluded by vertical occluders or randomly scattered dots (Orghian & Hidalgo, 2020).

This study revealed the impact of a social incident, that is, the COVID-19 epidemic, on perceptions of attractiveness of mask-worn faces. Given that larger attitude shifts regarding support for politicians concerned about climate change were found in individuals who reported greater suffering from hurricanes (Rudman et al., 2013), our finding that beliefs and perceptions regarding the attractiveness and healthiness of mask-worn faces had already changed just months after the explicit onset of the COVID-19 epidemic in Japan (the first patient was found on January 16) implies that the magnitude of the impact is large. Accordingly, we expect that modulation of the sanitary-mask effect directly reflects the progress of the epidemic. In other words, this study demonstrated a contextual modulation of facial perception that took place over a short period of time. However, given that the context in which individuals view target faces can modulate facial attractiveness in a laboratory setting (e.g., varying the proportion of beard-worn vs. clean-shaven faces; Janif et al., 2014), the modulation observed in this study may change with the severity of the epidemic. Long-term measurements of beliefs and perceptions regarding mask-worn faces would provide more information regarding the impact of the epidemic on societies worldwide.

There are two limitations to this study. First, the three-way Period × Baseline attractiveness × Mask presence interaction was not significant, probably due to differences in the baseline conditions of the previous (Miyazaki & Kawahara, 2016) and present studies, although the trend indicated by the results was consistent with the predicted direction. Therefore, the impact of the COVID-19 epidemic on perceived attractiveness warrants careful interpretation and further examination. Second, the change in the purpose of mask use might have introduced a demand bias effect such that participants might have avoided saying that they found a given mask-wearing woman unattractive due to the social norms governing mask-wearing. The demand bias may explain the results for faces with low attractiveness scores, but this explanation is not applicable to faces with high attractiveness scores. Therefore, we believe that the present results cannot be solely attributed to demand bias. Nonetheless, this is a limitation of this study, although it was unavoidable.
